# Cholera in United States Associated with Epidemic in Hispaniola

**DOI:** 10.3201/eid1711.110808

**Published:** 2011-11

**Authors:** Anna E. Newton, Katherine E. Heiman, Ann Schmitz, Tom Török, Andria Apostolou, Heather Hanson, Prabhu Gounder, Susan Bohm, Katie Kurkjian, Michele Parsons, Deborah Talkington, Steven Stroika, Lawrence C. Madoff, Franny Elson, David Sweat, Venessa Cantu, Okey Akwari, Barbara E. Mahon, Eric D. Mintz

**Affiliations:** Centers for Disease Control and Prevention, Atlanta, Georgia, USA (A.E. Newton, K.E. Heiman, A. Schmitz, T. Török, A. Apostolou, P. Gounder, K. Kurkjian, M. Parsons, D. Talkington, S. Stroika, B.E. Mahon, E.D. Mintz); Atlanta Research and Education Foundation Inc., Decatur, Georgia, USA (A.E. Newton, K.E. Heiman); Florida Department of Health, Tallahassee, Florida, USA (A. Schmitz, T. Török); New Jersey Department of Health and Senior Services, Trenton, New Jersey, USA (A. Apostolou); New York City Department of Health and Mental Hygiene, New York, New York, USA (H. Hanson, P. Gounder); Michigan Department of Community Health, Lansing, Michigan, USA (S. Bohm); Virginia Department of Health, Richmond, Virginia, USA (K. Kurkjian); Massachusetts Department of Public Health, Jamaica Plain, Massachusetts, USA (L.C. Madoff, F. Elson); North Carolina Division of Public Health, Raleigh, North Carolina, USA (D. Sweat); Texas Department of State Health Services, Austin, Texas, USA (V. Cantu); Houston Department of Health and Human Services, Houston, Texas, USA (O. Akwari)

**Keywords:** cholera, Vibrio cholerae, bacteria, Haiti, Dominican Republic, Hispaniola, United States, epidemic, expedited, dispatch

## Abstract

Cholera is rare in the United States (annual average 6 cases). Since epidemic cholera began in Hispaniola in 2010, a total of 23 cholera cases caused by toxigenic *Vibrio cholerae* O1 have been confirmed in the United States. Twenty-two case-patients reported travel to Hispaniola and 1 reported consumption of seafood from Haiti.

Cholera caused by toxigenic *Vibrio cholerae*, serogroup O1, serotype Ogawa, biotype El Tor, was confirmed on October 21, 2010, in Haiti and on October 31, 2010, in the Dominican Republic. These countries are on the island of Hispaniola. During October 21, 2010–April 4, 2011, >275,000 cholera cases and >4,700 deaths were reported from Hispaniola. Of these cases, 840 culture-confirmed cases and 10 deaths were reported from the Dominican Republic.

Illness caused by toxigenic *V. cholerae* O1 has been documented in the United States since 1832. During 1965–1991, an average of 5 cases per year were reported. During the Latin American cholera epidemic that started in 1991, the number of cholera cases in the United States increased because of importation of cases related to the epidemic to an average of 53 cases per year during 1992–1994 (*1**,**2*). As the Latin American epidemic waned, during 1995–2000, the average annual case count decreased to 10 (*3*). During 2000–2010, the average number of cases was 6, and 57% of case-patients had traveled internationally (*4*). This experience raised concern that a dramatic increase in US cholera cases could result from the Hispaniola epidemic.

In the United States, cholera is confirmed by identification of toxigenic *V. cholerae* serogroup O1 or O139 or by serologic evidence of infection in a patient with diarrhea and an epidemiologic link to a culture-confirmed case. Since 2000, suspected *V. cholerae* isolates have been sent by state public health laboratories to the Centers for Disease Control and Prevention (CDC) for confirmation and characterization.

We summarize characteristics of confirmed US cases associated with the Hispaniola epidemic that were reported to the CDC Cholera and Other *Vibrio* Illness Surveillance System, a national database of all laboratory-confirmed cholera and vibriosis cases. For each case of cholera, state and local health officials submit a Cholera and Other *Vibrio* Illness Surveillance System report form that contains demographic, clinical, and epidemiologic information, including selected food and water exposures associated with cholera, travel history, and vaccination status.

## The Study

**Figure 1:**
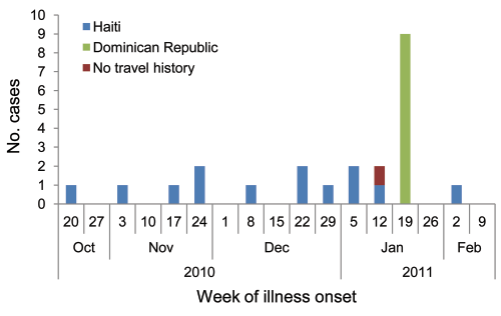
Confirmed cholera cases (n = 23), by onset date and travel history, United States, October 21, 2010–February 4, 2011.

**Figure 2:**
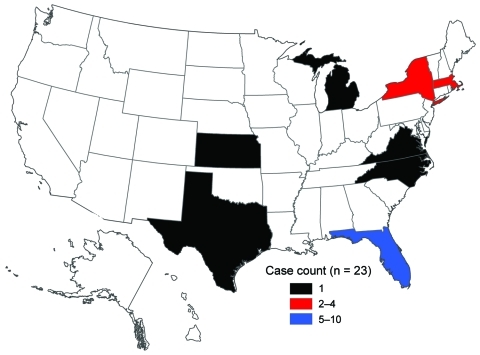
Geographic distribution of cholera cases in the United States associated with Hispaniola, October 21, 2010–April 4, 2011.

The first US case associated with the epidemic in Hispaniola was laboratory confirmed on November 15, 2010, in a US resident who had traveled to Haiti and returned to Florida. The first case in a patient with history of travel to Dominican Republic was laboratory confirmed on January 29, 2011. As of April 4, 2011, a total of 23 cholera cases associated with the Hispaniola epidemic had been confirmed (Figure 1). Patients resided in Florida (10), Massachusetts (4), New York City (4), Kansas (1), Michigan (1), North Carolina (1), Virginia (1), and Texas (1) (Figure 2). Illness onset dates ranged from October 23, 2010, to February 2, 2011. Median age was 38 years (range 9–84 years), and 43% were female patients.

All patients were treated with antimicrobial agents, rehydration, or both; 9 (39%) were hospitalized, 6 (30%) sought care at an emergency department, and none died. Six patients had illness onset before returning to the United States, 5 had illness onset on the day of return, and 12 had illness onset 1–11 days after return (typical incubation period for cholera is 18 hours–5 days) (*5*).

Twenty cases were culture confirmed and 3 were confirmed by serologic testing. All 20 isolates matched the Haiti isolate outbreak pattern by pulsed-field gel electrophoresis. Susceptibility results for antimicrobial drug tested showed that all isolates were resistant to trimethoprim/sulfamethoxazole, furazolidone, nalidixic acid, sulfisoxazole, and streptomycin, and 18 isolates showed intermediate resistance to chloramphenicol, ampicillin, or amoxicillin/clavulanic acid.

Thirteen patients reported recent travel to Haiti (median length of stay 7 days, range 2–54 days) and 9 to Dominican Republic (median length of stay 4 days, range 2–9 days). One patient reported no recent travel but consumed cooked conch brought to the United States from Haiti by relatives. Travel was reported to the following departments in Haiti: Artibonite (2), Ouest (7), Centre (1), Nord (1), and Sud (1). One case-patient traveled to 2 departments, and 2 did not specify a destination. All case-patients associated with the Dominican Republic had attended a wedding in La Romana Province on January 22, 2010; an investigation conducted by the Dominican Republic Ministry of Health is ongoing. Aside from 2 case-patients who traveled to this wedding together, no other case-patients reported traveling together.

Visiting friends or relatives was the main reason for travel to Haiti (Table 1). Four patients traveled to Haiti to participate in relief activities, 2 as medical volunteers, 1 on a mission trip, and 1 to distribute canned foods. One patient immigrated to the United States from Haiti during the incubation period. A wide range of exposures was reported (Table 2); 5 patients were exposed to persons with cholera or cholera-like illness and to other risk factors for cholera acquisition. Medical volunteers participated in direct patient care. One volunteer reported no apparent lapses in safe water and food practices, although detailed information about food preparation was not available. No additional information was available for the other volunteer.

**Table 1:** Reason for travel by destination for 22 cholera patients, United States, October 21, 2010–February 4, 2011*

Reason for travel	Haiti	Dominican Republic
To visit relatives or friends	7	9
Business	2	0
Aid travel	4	0
Immigration to United States	1	0

*Persons may have traveled for >1 reason.

**Table 2:** Selected exposures during 4 d (7 d for body of water exposure) before illness onset in 23 cholera patients, United States, October 21, 2010–February 4, 2011*

Exposure	No. persons exposed	No. case-patients with information available
Foodborne		
Street-vended food	1	20
Cooked seafood	9	20
Raw seafood	3	18
Waterborne		
Any body of water†	7	20
Swimming/bathing	3	6
Well water (drinking)	1	1
Other		
Seafood handled	1	7
Person(s) with cholera or cholera-like illness	5	19

*Exposures were not investigated further. †Water exposures include local lake and stream of water running down a street in Haiti; a pool in the Dominican Republic; ocean (Dominican Republic); unspecified location in Port-au-Prince, Haiti; and a tank at a medical center in Haiti. Two case-patients did not specify body of water exposure.

Seven of 15 patients with information available reported receiving cholera prevention information before travel. Sources included newspaper articles (4), friends (4), CDC traveler’s hotline (1), and the World Health Organization website (1); 2 patients reported >1 source. None had ever received cholera vaccine. Two patients reported receiving a Travel Health Alert Notice upon arrival in the United States (M. Selent, unpub. data).

## Conclusions

Six months after the Hispaniola cholera epidemic started in Haiti, 23 associated cases were recognized in the United States. All cases were associated with recent travel to Hispaniola or with consumption of seafood from Haiti. The risk for cholera transmission in the United States is low because of improved water and sanitation, and there is no evidence of secondary transmission. Florida, New York, and Massachusetts have the highest populations of persons of Haitian or Dominican ancestry (*6*). Most cases were reported from Florida, the state with the largest Haitian population. However, case-patients also resided in states with small Haitian and Dominican populations. Travel between the United States and Haiti is straightforward; 4 US airports offer daily direct flights from Florida and New York to Port-au-Prince. Many persons, including many of Haitian descent, traveled from the United States to Haiti to help with the response to the January 2010 earthquake in Port-au-Prince.

Person-to-person transmission of cholera has only rarely been reported; cases in medical workers are almost always attributable to consumption of contaminated food or water. Person-to-person transmission is not clearly supported for either of the cases we report in medical workers, although it cannot be ruled out. Continued surveillance and detailed investigation of cases in medical workers is warranted to further define the risk, if any, of person-to-person transmission.

Echoing the Latin American cholera epidemic in the 1990s, the number of US cholera cases has increased after the cholera epidemic in Hispaniola. Travelers to cholera-affected areas should be aware of the risk and should follow prevention measures to avoid infection. In particular, travelers visiting friends or relatives may be at higher risk for travel-associated infection (*7*). Few case-patients had received cholera prevention education (educational materials available at www.cdc.gov/cholera/index.html); no cholera vaccine is licensed in the United States. Until cholera in Haiti and Dominican Republic resolves, clinicians, microbiologists, and public health workers in the United States should be prepared for more cases in travelers returning from Hispaniola.
